# Association of Body-Weight Fluctuation With Outcomes in Heart Failure With Preserved Ejection Fraction

**DOI:** 10.3389/fcvm.2021.689591

**Published:** 2021-06-14

**Authors:** Yi Li, Yuan Yu, Yuzhong Wu, Weihao Liang, Bin Dong, Ruicong Xue, Yugang Dong, Wengen Zhu, Peisen Huang

**Affiliations:** ^1^Department of Cardiology, The First Affiliated Hospital of Sun Yat-sen University, Guangzhou, China; ^2^National Health Commission Key Laboratory of Assisted Circulation, Sun Yat-sen University, Guangzhou, China; ^3^National-Guangdong Joint Engineering Laboratory for Diagnosis and Treatment of Vascular Diseases, Guangzhou, China; ^4^Guangdong Provincial People's Hospital, Guangdong Academy of Medical Sciences, Guangzhou, China; ^5^Department of Cardiology, Guangdong Cardiovascular Institute, Guangzhou, China

**Keywords:** heart failure with preserved ejection fraction, body weight, fluctuation, outcome, heart failure

## Abstract

**Aims:** To investigate the relationship between body-weight fluctuation and risks of clinical outcomes in patients with heart failure with preserved ejection fraction (HFpEF).

**Methods and Results:**We measured intra-individual variations in body weight from baseline and follow-up visits in 1,691 participants with HFpEF from the Americas from the Treatment of Preserved Cardiac Function Heart Failure with an Aldosterone Antagonist (TOPCAT) trial. The primary endpoint was any cardiovascular events (a composite of death from cardiovascular disease, non-fatal myocardial infarction, aborted cardiac arrest, or hospitalization for HF). The body-weight fluctuation was measured according to average successive variability and high variability was defined as greater than or equal to the median. After adjustment for risk factors, mean body weight and weight change, each increase of 1 standard deviation in body-weight variability was significantly associated with increased risks of any cardiovascular events (hazard ratio [HR] 1.23, 95% confidence interval [CI] 1.15–1.33, *P* < 0.001). Patients with high variability had a 47% increased risk of any cardiovascular events and 27% increased risk of all-cause death compared with those with low variability. Such association was similar among patients with New York Heart Association functional class I/II vs. III/IV, obesity vs. non-obesity, and weight loss, gain vs. stability (the *P*-values for interaction were all insignificant).

**Conclusion:** Among patients with HFpEF, body-weight fluctuation was associated with increased risks of cardiovascular events independent of traditional cardiovascular risk factors, and regardless of HF severity, baseline weight or weight change direction.

**Clinical Trial Registration:** Aldosterone antagonist therapy for adults with heart failure and preserved systolic function (TOPCAT), https://clinicaltrials.gov, identifier [NCT00094302].

## Introduction

Heart failure (HF) is a global pandemic affecting at least 26 million people worldwide and is increasing in prevalence (1). Body weight of patients with HF often oscillates over time, and fluctuations in weight may have negative consequences. Monitoring of body weight has been recommended in self-care for all patients by HF management guidelines (2, 3). The relationship between body weight and outcomes is complex in patients with established HF (4–7). A U-shaped distribution curve has been proved in which mortality is greatest in under-weight patients, lower in normal to overweight patients, and higher again in more severely obese patients with HF (8). Weight loss may reflect cachexia status in advanced HF (9), and associates with a higher risk of mortality and cardiovascular events (10–14). Weight gain is also associated with a modestly increased mortality risk (11, 13). However, the association between body-weight fluctuation and health outcomes in patients with HF is not yet fully established. Furthermore, whether HF severity, body weight at baseline, and direction of weight change affect the association of body-weight fluctuation and outcomes also remains unknown.

Accordingly, based on the data from the TOPCAT (Treatment of Preserved Cardiac Function Heart Failure with an Aldosterone Antagonist) trial, which involved patients with established HF with preserved ejection fraction (HFpEF), we performed a *post-hoc* analysis to explore the relationship between intra-individual fluctuations in body weight and the risk of cardiovascular events. We further explored the interaction between body-weight fluctuation and HF severity, baseline weight, and weight change direction.

## Methods

### Study Population

We conducted a *post-hoc* analysis of the TOPCAT trial, a multi-center, international, randomized, double-blind, placebo-controlled trial of spironolactone in adults with HFpEF recruited from over 270 clinical sites. The design of the TOPCAT trial was described in detail previously (15). The primary results of the trial were published at NEJM.org (16). In the present study, we included patients from the Americas enrolled in the TOPCAT trial, who had at least two post-baseline measurements of body weight. Data on vital signs, including body weight and height, were collected at baseline. Patients were followed at 1, 2, 4, 8,12, and 18 months, and every half year thereafter, at which times data on vital signs, including body weight, were collected. Patients were followed for a mean of 3.5 years.

The TOPCAT trial was funded by the National Heart, Lung, and Blood Institute as a contract with the Brigham and Women's Hospital (Clinical Coordinating Center) and the New England Research Institute (Data Coordinating Center). All study participants provided written informed consent. We acquired the dataset of the TOPCAT trial from the National Heart, Lung, and Blood Institute (NHLBI) by applying to Biologic Specimen and Data Repository Information Coordinating Center (BIOLINCC, https://biolincc.nhlbi.nih.gov/). Our study was approved by the Medical Ethical Committee of the First Affiliated Hospital, Sun Yat-sen University. The TOPCAT investigators were not involved in the present study.

### Measures of Body-Weight Variability

Body-weight variability was assessed using three indices: (1) standard deviation (SD), (2) variability independent of the mean (VIM), and (3) average successive variability (ASV). VIM was calculated as 100 × SD/mean β, where β is the regression coefficient, based on the natural logarithm of the SD over the natural logarithm of the mean. In this study, ASV was used as the primary variability measure, defined as the average absolute difference between successive values.

### Study Outcomes

The primary outcome was the occurrence of any cardiovascular events (a composite of death from cardiovascular disease, non-fatal myocardial infarction, aborted cardiac arrest, or hospitalization for HF). The secondary outcomes were individual components of the primary outcome, as well as all-cause death, myocardial infarction, and new onset of atrial fibrillation.

### Statistical Analysis

We stratified patients into two groups based on the body-weight variability: high variability (greater than or equal to the median of ASV) and low variability (below the median of ASV). Categorical variables were described by frequencies with percentages, and continuous variables were described by a median with interquartile ranges. Demographic and clinical characteristics were compared between the two groups of high vs. low variability. Kruskal-Wallis test for continuous variables and chi-squared tests for categorical variables.

The relation between body-weight variability and the risk of outcomes was evaluated with the use of body-weight variability as both continuous and categorical variables. When analyzed as a categorical variable, the Kaplan-Meier survival analysis and Cox proportional hazards models were performed to evaluate the risk of outcomes between groups of high vs. low variability. When analyzed as a continuous variable, Cox proportional hazards models were used to estimate hazard ratios (HRs) and 95% confidence intervals (CIs) for outcomes associated with per increase in variability of 1 SD. Four models were used: model 1 being unadjusted; model 2 adjusting model 1 for diuretic therapy at baseline; model 3 adjusting model 2 for mean body weight and change in weight, taking directionality into account (continuous variable); and model 4 adjusting model 3 for + age, sex, race, smoking status, diabetes, atrial fibrillation, peripheral arterial disease, previous hospitalization for HF, prior myocardial infarction, known stroke, chronic obstructive pulmonary disease, New York Heart Association (NYHA) class, systolic blood pressure, heart rate, ejection fraction, estimated glomerular filtration rate, and number of weight measurement, with stepwise selection of covariates which were significant at the 0.05 level. Sensitivity analyses were conducted using other measures of variability (±SD and VIM) to evaluate the consistency of the results.

Subgroup analyses were performed to evaluate whether the relationship between body-weight variability and outcomes differed by sex, baseline NYHA class, body-mass index (BMI), and weight change direction, by introducing a weight variability × variable interaction terms. Patients were assigned to the following subgroups: (1) men or women; (2) NYHA I/II or NYHA III/IV; (3) obesity (BMI, ≥30 kg/m^2^) or non-obesity (BMI, <30 kg/m^2^); (4) weight loss (weight witnessed a decrease of ≥5%), weight gain (weight witnessed an increase of ≥5%), or weight stability (weight change <5%). Unadjusted and adjusted models were constructed to evaluate the association of high variability in weight and the risk of the primary outcome in the above-mentioned subgroups.

All statistical analyses were conducted using SAS statistical software version 9.4 (SAS Institute Inc) and the survival plot was made using GraphPad Prism 7. All comparisons were 2-sided and *P* < 0.05 was considered statisticall*y* significant.

## Results

### Characteristics of the Patients

Among all study populations enrolled in the trial, 1,691 participants met the inclusion criteria for the present analysis. The median age was 72 years (IQR 64–79), and 49.5% were. The median baseline body weight of the patients was 90.7 kg (IQR 76.0–108.9). The median number of weight measures was 7 (range, 2–11) (Supplementary Figure 1A in the Supplementary Appendix). The median body-weight variability was 2.1 kg (IQR 1.4–3.1) (Supplementary Figure 1B in the Supplementary Appendix). The median body-weight variability was 3.1 kg (IQR 2.5–4.1) and 1.4 kg (IQR 1.0–1.7) for patients in high and low variability group. Table 1 outlines the baseline characteristics of the study population with high- vs. low-weight variability. Compared with patients with low variability, those with high variability were younger, predominantly males, less likely to be white, had higher proportions of previous HF hospitalization or myocardial infarction, chronic obstructive pulmonary disease, and diabetes mellitus. They also had more often with NYHA class III/IV, and had higher baseline body weight.

**Table 1:** Characteristics of the patients by body-weight variability groups.

	**Low variability**	**High variability**	**Total**	***P-*value**
	***N* = 842**	***N* = 849**	***N* = 1,691**	
**Demographic**				
Age, median (IQR), y	75 (68–81)	69 (62–77)	72 (64–79)	<0.001
Women, *n* (%)	459 (54.5)	378 (44.5)	837 (49.5)	<0.001
Race, *n* (%)				<0.001
White	690 (81.9)	646 (76.1)	1,336 (79.0)	
Black	103 (12.2)	177 (20.8)	280 (16.6)	
**Clinical**				
Randomization to spironolactone, *n* (%)	421 (50.0)	431 (50.8)	852 (50.4)	0.753
Diuretics, *n* (%)	740 (87.9)	768 (90.5)	1,508 (89.2)	<0.001
Current smoker, *n* (%)	45 (5.34)	61 (7.18)	106 (6.3)	0.18
Previous hospitalization for CHF, *n* (%)	442 (52.5)	556 (65.5)	998 (59.0)	<0.001
Previous myocardial infarction, *n* (%)	153 (18.2)	197 (23.2)	350 (20.7)	0.011
Known stroke, *n* (%)	68 (8.1)	85 (10.0)	153 (9.0)	0.168
COPD, *n* (%)	123 (14.6)	161 (19.0)	284 (16.8)	0.017
Hypertension, *n* (%)	760 (90.3)	762 (89.7)	1,522 (90.0)	0.672
Peripheral Arterial Disease, *n* (%)	90 (10.7)	112 (13.2)	202 (11.9)	0.115
Atrial fibrillation, *n* (%)	384 (45.6)	338 (39.8)	722 (42.7)	0.015
Diabetes mellitus, *n* (%)	305 (36.2)	449 (52.9)	754 (44.6)	<0.001
Previous pacemaker, *n* (%)	126 (15.0)	108 (12.7)	234 (13.8)	0.178
Previous ICD, *n* (%)	17 (2.0)	25 (2.9)	42 (2.5)	0.223
NYHA class III/IV, *n* (%)	232 (27.5)	355 (41.8)	587 (34.7)	<0.001
Heart rate, median (IQR), (bpm)	67 (60–75)	69 (62–76)	68 (61–76)	<0.001
SBP, median (IQR), (mmHg)	128 (118–138)	128 (118–139)	128 (118–138)	0.638
Body weight, median (IQR), (Kg)	82.3 (71.2–95.7)	101.6 (85.3–121.0)	90.7 (76.0–108.9)	<0.001
Ejection fraction, median (IQR)	60 (53–65)	58 (52–64)	58 (53–64)	0.177
eGFR, median (IQR)	61.8 (49.5–76.6)	60.4 (48.9–77.2)	61.3 (49.0–77.0)	0.778

*CHF, chronic heart failure; COPD, chronic obstructive pulmonary disease; ICD, implantable cardioverter defibrillator; NYHA, New York Heart Association; SBP, systolic blood pressure; eGFR, estimated glomerular filtration rate*.

### Body-Weight Variability and Outcomes

When body-weight variability (as measured by ASV) was used as a continuous variable in the adjusted model 4, each increase in body-weight variability of 1 SD (1.88 kg) was associated with increased risks of any cardiovascular events (HR 1.23, 95% CI 1.15–1.33, *P* < 0.001), non-fatal myocardial infarction (HR 1.30, 95% CI 1.09–1.55, *P* = 0.004), hospitalization for HF (HR 1.28, 95% CI 1.19–1.38, *P* < 0.001) (Table 2). Sensitivity analyses with two other indices of variability (SD, VIM) observed a consistent association between body-weight variability and risk of any cardiovascular events (Supplementary Table 1 in the Supplementary Appendix).

**Table 2:** Risk of outcomes in per 1-SD change of body-weight variability.

**Outcomes**	Model 1^*^		**Model 2**^**#**^		**Model 3**^****§****^		**Model 4**^****¶****^	
	**HR (95% CI)**	***P*-value**	**HR (95% CI)**	***P*-value**	**HR (95% CI)**	***P*-value**	**HR (95% CI)**	***P*-value**
Any cardiovascular events	1.27 (1.19–1.35)	<0.001	1.27 (1.19–1.35)	<0.001	1.35 (1.25–1.45)	<0.001	1.23 (1.15–1.33)	<0.001
Cardiovascular death	0.97 (0.84–1.13)	0.738	0.96 (0.83–1.12)	0.633	1.11 (0.95–1.30)	0.184	1.00 (0.88–1.14)	0.980
Myocardial infarction	1.13 (0.95–1.35)	0.153	1.13 (0.95–1.35)	0.156	1.26 (1.05–1.52)	0.013	1.30 (1.09–1.55)	0.004
Hospitalization for HF	1.36 (1.28–1.45)	<0.001	1.36 (1.27–1.45)	<0.001	1.41 (1.30–1.52)	<0.001	1.28 (1.19–1.38)	<0.001
All-cause death	1.10 (1.00–1.21)	0.045	1.09 (0.99–1.20)	0.068	1.22 (1.10–1.35)	<0.001	1.05 (0.95–1.14)	0.332
New onset atrial fibrillation	1.16 (0.98–1.37)	0.074	1.15 (0.98–1.36)	0.092	1.12 (0.92–1.36)	0.275	1.09 (0.92–1.29)	0.306

* *Model 1 was unadjusted;*

# *Model 2 was adjusted for diuretics*.

§ *Model 3 was adjusted for diuretics, mean body weight, and change in weight, taking directionality into account;*

¶ *Model 4 was adjusted for the same variables as model 3 and for age, sex, race, smoking status, diabetes status, trial fibrillation, peripheral arterial disease, previous hospitalization for chronic heart failure, prior myocardial infarction, known stroke, chronic obstructive pulmonary disease, New York Heart Association class, systolic blood pressure, heart rate, ejection fraction, estimated glomerular filtration rate, number of weight measurement. HF, heart failure; HR, hazard ratio; CI, confidence interval*.

**Figure 1:**
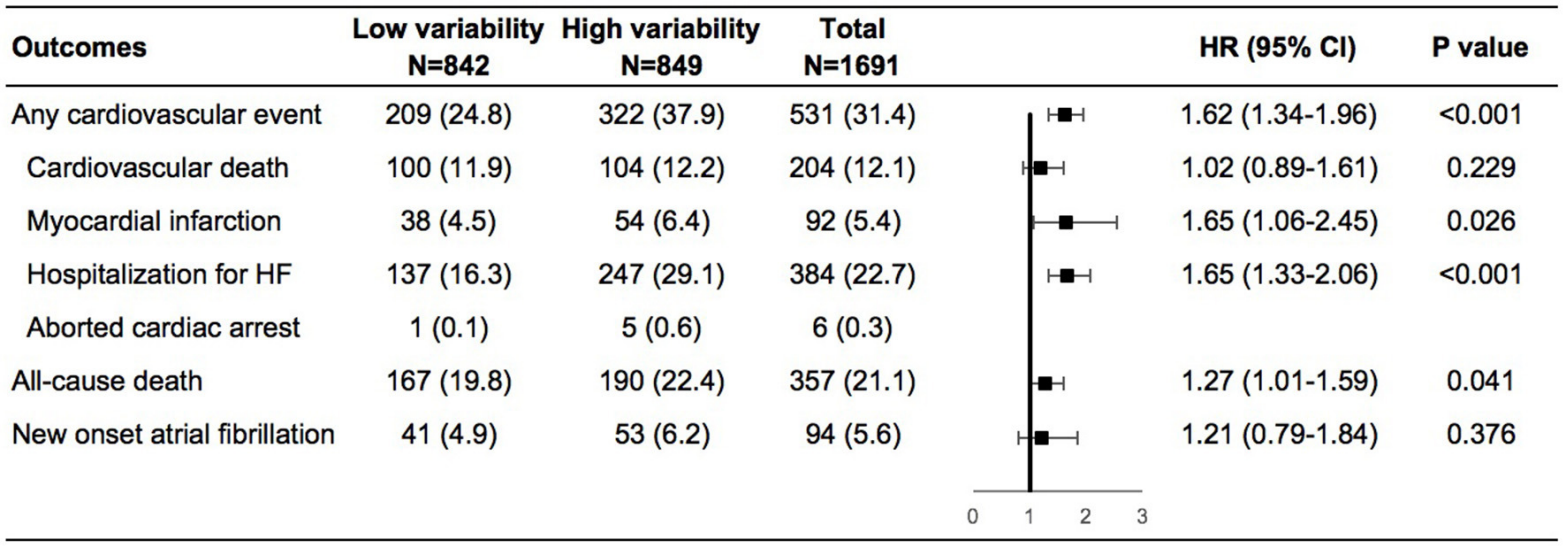
Risk of outcomes in the high vs. low body-weight variability in multivariable model. The multivariable model was adjusted for diuretics, mean body weight, change in weight, age, sex, race, smoking status, diabetes status, atrial fibrillation, peripheral arterial disease, previous hospitalization for chronic heart failure, prior myocardial infarction, known stroke, chronic obstructive pulmonary disease, New York Heart Association class, systolic blood pressure, heart rate, ejection fraction, estimated glomerular filtration rate, number of weight measurement. HF, heart failure; HR, hazard ratio.

During a mean follow-up of 3.5 years, cardiovascular events occurred in 209 (24.8%) and 322 (37.9%) of patients with low and high weight variability, respectively (Supplementary Figure 2 in the Supplementary Appendix). In the adjusted model 4, compared with patients with low-weight variability, those with high-weight variability had an increase in the risks of any cardiovascular events by 62%, non-fatal myocardial infarction by 65%, and HF hospitalization by 65%, all-cause death by 27%, and a non-significant increase in the risk of new onset atrial fibrillation of 21% (Figure 1).

### Subgroup Analyses

**Figure 2:**
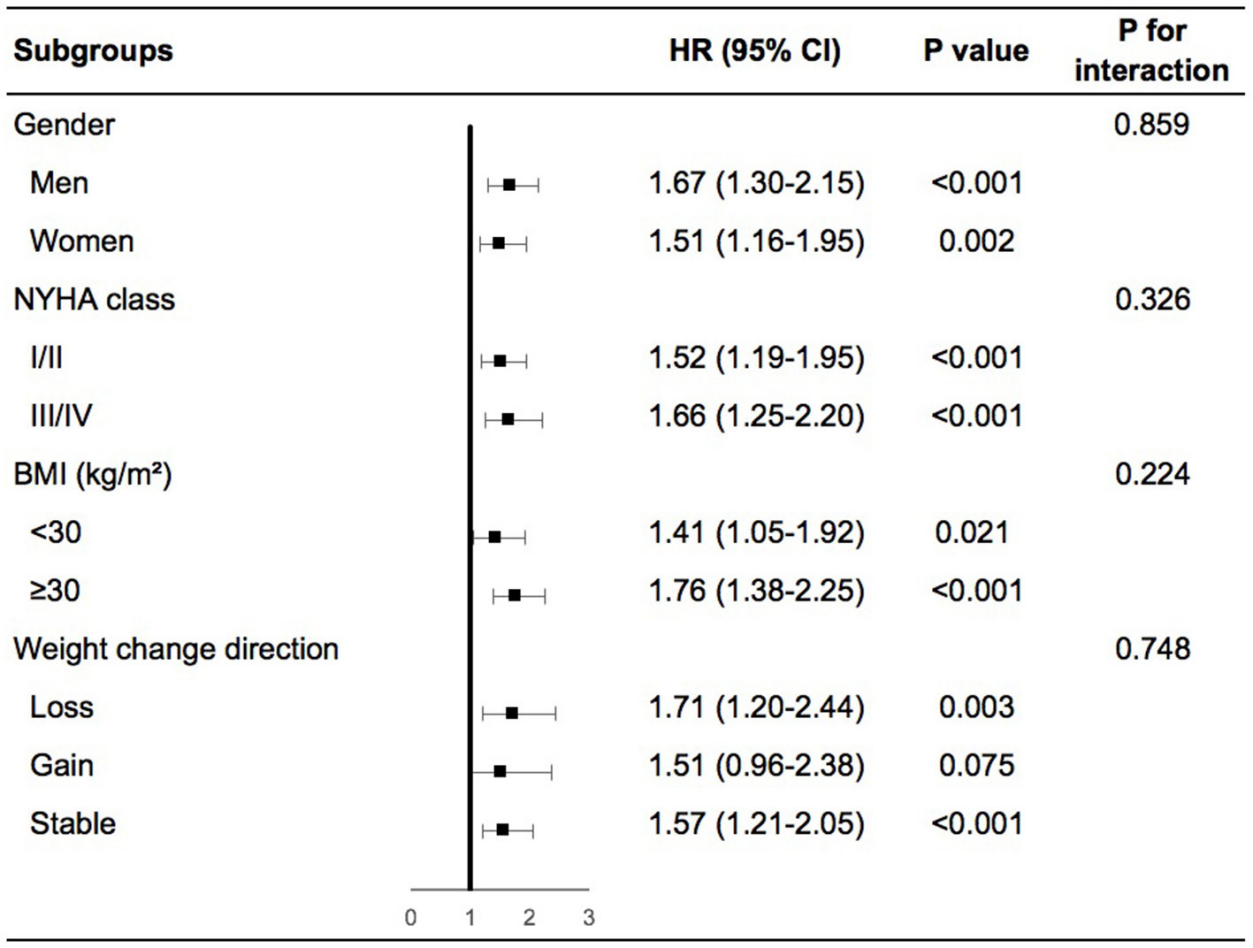
Body-weight variability and risk of any cardiovascular events for various subgroups in multivariable model. The multivariable model was adjusted for diuretics, mean body weight, change in weight, age, sex, race, smoking status, diabetes status, atrial fibrillation, peripheral arterial disease, previous hospitalization for chronic heart failure, prior myocardial infarction, known stroke, chronic obstructive pulmonary disease, NYHA class, systolic blood pressure, heart rate, ejection fraction, estimated glomerular filtration rate, number of weight measurement. NYHA, New York Heart Association; BMI, body mass index; HR, hazard ratio.

Patients with high variability in body weight had significant higher risk of any cardiovascular events than patients with low variability in various subgroups including men or women, NYHA class I/II or III/IV at baseline, obesity, or non-obesity, weight loss, gain or stability during the follow-up period (Supplementary Figures 3A–D in the Supplementary Appendix). In the adjusted model, similar findings were demonstrated except for patients with non-obesity, in which high variability in body weight was associated with a numerically increased risk of any cardiovascular events although not significant (Figure 2). We further evaluated the interactions on any cardiovascular events between body-weight variability and the subgroups based on gender, baseline NYHA class, BMI, and weight change direction. As shown in Figure 2, none of these tests for interactions were statistically significant (the *P*-values were 0.86, 0.33, 0.22, and 0.75, respectively).

## Discussion

In this *post-hoc* analysis of patients with established HFpEF who participated in the TOPCAT trial, fluctuation in body weight was strongly associated with the risk of cardiovascular events and even death independent of traditional risk factors. Moreover, the associations observed were consistent among those who were at NYHA class I/II or III/IV, non-obese or obese, weight loss, gain, or stability over time.

Prior studies have explored the complex impact of baseline weight and weight change on outcomes in patients with HF. The “obesity paradox” (4–6) that more favorable prognosis in obese vs. normal-weight patients was found. Moreover, both weight loss and weight gain were associated with poor prognosis (10–14). However, another important aspect of the body weight, the variability over time (17, 18), has not been evaluated in HF. Highly variable body-weight was associated with increased total mortality and morbidity (19) and a higher incidence of HF (20) and diabetes mellitus (21) in the general population. Other studies found body-weight variability was associated with increased risks of cardiovascular events and mortality in patients with coronary artery disease (22) and type 2 diabetes (23–25). To the best of our knowledge, this analysis is the first one to demonstrate that in patients with HF, body-weight variability was also independently associated with a significant increase in the risk of cardiovascular events and death. A prior study found body-weight fluctuation was associated with increased risk of incident atrial fibrillation in the general population (26). We proved that this association also existed in patients with HF.

Fluctuation in body weight is a common phenomenon, especially in patients with HF. Weight loss is commonly prescribed as a lifestyle intervention in obese patients. However, weight loss is frequently followed by weight gain (or “weight cycling”) or by other patterns of weight fluctuation. In patients with established HF, weight loss may also be caused by the higher total energy expenditure of HF in cachexia status, and rapid weight gain often appears when volume overloaded. Prior studies (27, 28) suggested simply discharge education including monitoring of body weight can improve clinical outcomes. Thus, both the American College of Cardiology/American Heart Association (2) and European Society of Cardiology (3) guidelines for HF recommend patients with HF should receive specific education to facilitate self-care, including weight monitoring. Whereas, to what extend that weight fluctuation affects HF prognosis is not known. We found that in patients with HFpEF, each 1-SD increase in body-weight variability increased the risk of any cardiovascular events by 30% and the risk of mortality by 25%. The mechanism behind such association remains unclear. In this analysis, a higher risk of new onset atrial fibrillation associated with body-weight variability may lead to acute decompensation and hospitalization for HF. The association between increased body weight variability and adverse cardiovascular events and mortality highlights the substantial importance of avoiding weight fluctuation in long-term HF care.

The associations observed in our study may be due to reasons other than causality. Moreover, higher body-weight fluctuation may be a marker of advanced HF that has a worse prognosis. However, patients with NYHA class I/II witnessed similar results as patients with NYHA class III/IV in our study. Obvious weight loss due to chronic wasting may also proceed worse prognosis. However, patients with weight loss, gain, or stability yielded similar results, suggested the association was independent of weight change direction. Prior studies reported significant interactions with BMI in the association between weight variability and outcomes in patients with coronary artery disease (22) or type 2 diabetes (23). However, such interaction was not found in our study.

### Study Limitations

The present study has certain limitations as follows. First, we acknowledge that the main aim of the TOPCAT Trial was not to determine the role of weight variation in patients with HFpEF, thus, future studies of weight variation targets may be warranted. Second, we could not determine whether the weight change was intentional or unintentional, which may have different effects on prognosis. Third, body weight was collected at certain points, variability calculated may not reflect the whole follow-up phase. Fourth, because the analytic sample was limited to patients with HFpEF with stringent inclusion and exclusion criteria, additional studies in a broad spectrum of patients with HF are required to generalize our results. Fifth, since participants were enrolled more than 10 years ago in the TOPCAT trial, this analysis may not reflect current state of art.

## Conclusions

In patients with HFpEF, body-weight fluctuations were independently associated with a significant increase in the risk of cardiovascular events. The magnitude of this risk increased with greater variability in body weight and was independent of HF severity, baseline weight, or direction of weight change.

## Data Availability Statement

The datasets presented in this study can be found in online repositories. The names of the repository/repositories and accession number(s) can be found in the article/Supplementary Material.

## Ethics Statement

The studies involving human participants were reviewed and approved by the ethics committee of the First Affiliated Hospital of Sun Yat-sen University. The patients/participants provided their written informed consent to participate in this study.

## Author Contributions

PH, WZ, and YD design the research. YL, YY, YW, WL, BD, and RX analyse the data. YL, YY, PH, and WZ write the article. All authors contributed to the article and approved the submitted version.

## Conflict of Interest

The authors declare that the research was conducted in the absence of any commercial or financial relationships that could be construed as a potential conflict of interest.
