# Supplementary material for: Association of Body-Weight Fluctuation With Outcomes in Heart Failure With Preserved Ejection Fraction
Source: Front Cardiovasc Med. 2021 Jun 14;8:689591. doi: 10.3389/fcvm.2021.689591 (PMC8236532; doi:10.3389/fcvm.2021.689591)
Supplement: Supplementary file 1 [file Data_Sheet_1.pdf]

Supplementary appendix

Figure S1a Frequency Distribution of Number of Body Weight Measurements.

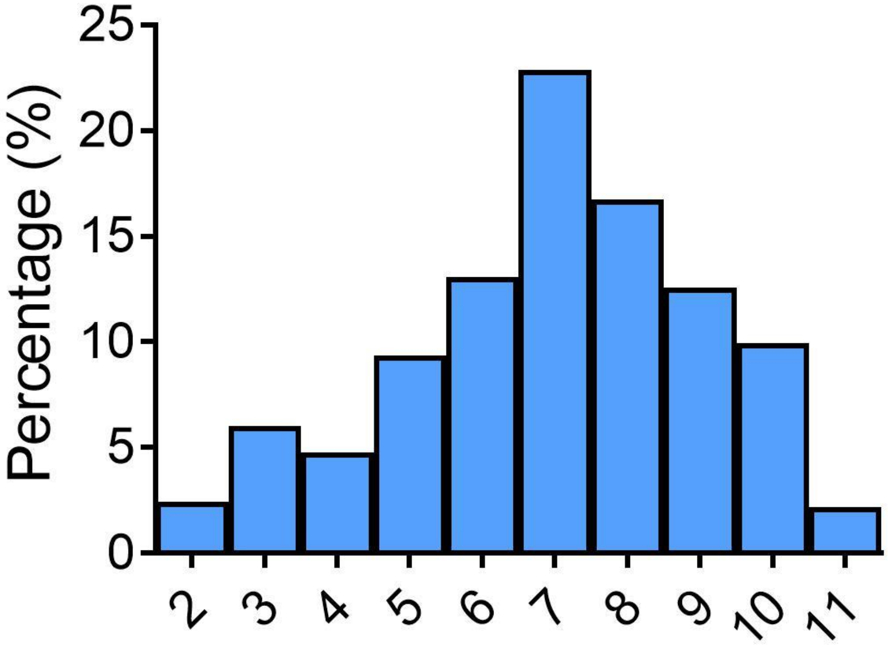

Figure S1b Frequency Distribution of Body Weight Variability.

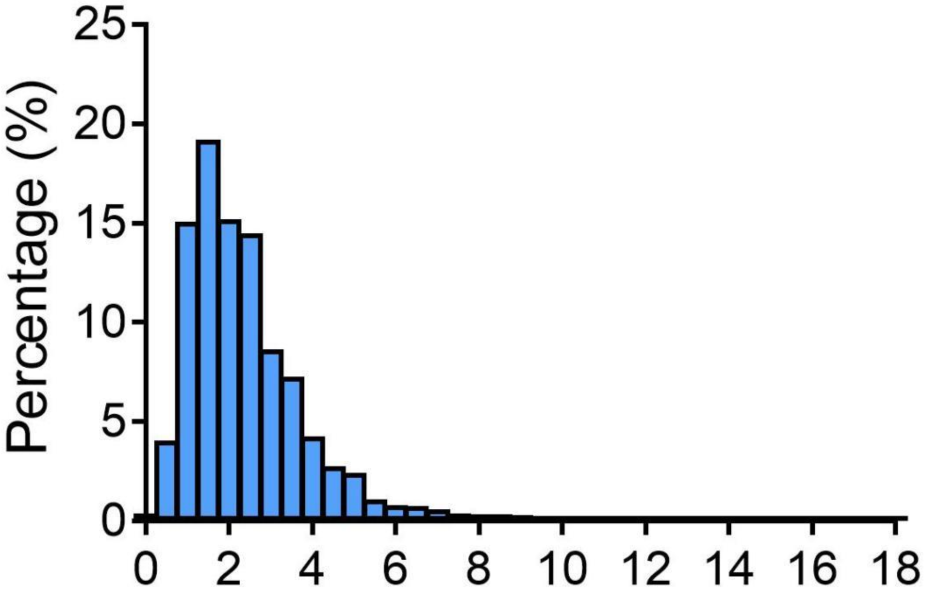

Figure S2 Time to any cardiovascular events by body-weight variability groups.

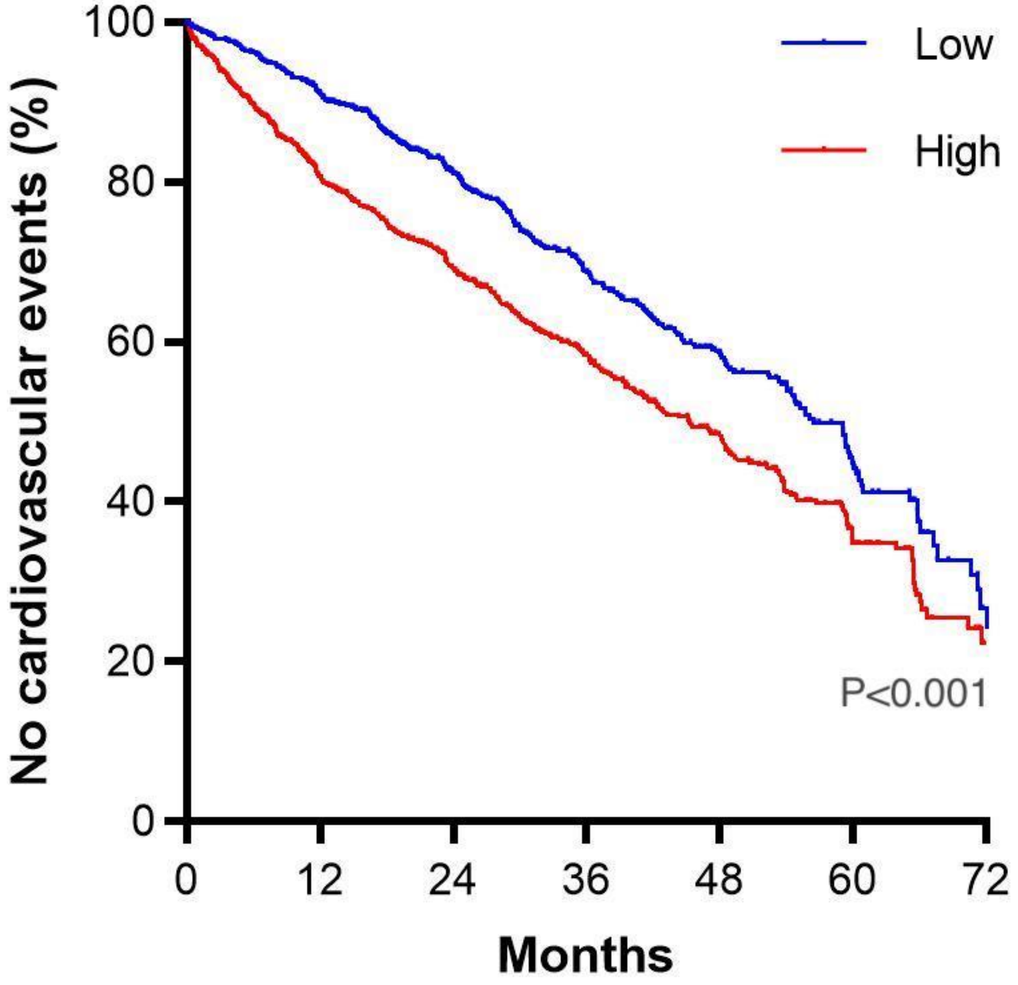

Table S1 Sensitivity analysis: risk of any cardiovascular events in per 1-SD change of body-weight variability.

**Variability measure**	**Model 1***		**Model 2#**		**Model 3§**		**Model 4¶**	
	HR (95% CI)	P value	HR (95% CI)	P value	HR (95% CI)	P value	HR (95% CI)	P value
Standard deviation	1.14 (1.06-1.22)	<0.001	1.16 (1.07-1.25)	<0.001	1.17 (1.08-1.27)	<0.001	1.18 (1.09-1.27)	<0.001
Variability independent of the mean	1.16 (1.07-1.25)	<0.001	1.13 (1.06-1.21)	<0.001	1.17 (1.07-1.27)	<0.001	1.18 (1.09-1.27)	<0.001

Per 1-SD change was 2.57 and 2.29 for standard deviation and variability independent of the mean, respectively.

* Model 1 was unadjusted.

# Model 2 was adjusted for diuretics.

§ Model 3 was adjusted for diuretics, mean body weight and change in weight, taking directionality into account.

¶ Model 4 was adjusted for the same variables as model 3 and for age, sex, race, smoking status, diabetes status, atrial fibrillation, peripheral arterial disease, previous hospitalization for chronic heart failure, prior myocardial infarction, known stroke, chronic obstructive pulmonary disease, New York Heart Association class, systolic blood pressure, heart rate, ejection fraction, estimated glomerular filtration rate, number of weight measurement.

HR, hazard ratio; CI, confidence interval.

Figure S3a Body-weight variability and risk of any cardiovascular events by gender.

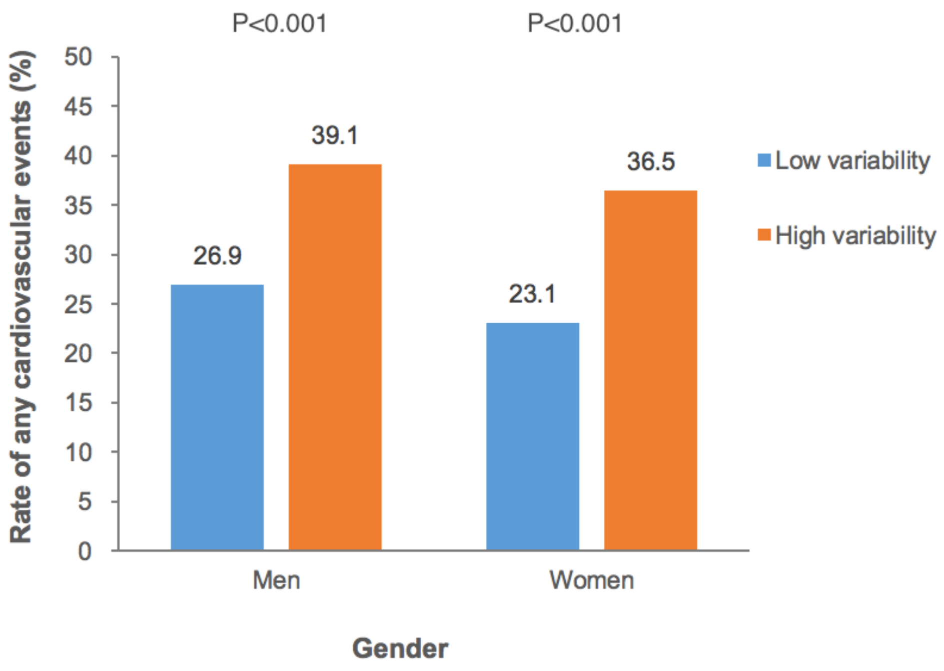

Figure S3b Body-weight variability and risk of any cardiovascular events by baseline NYHA class.

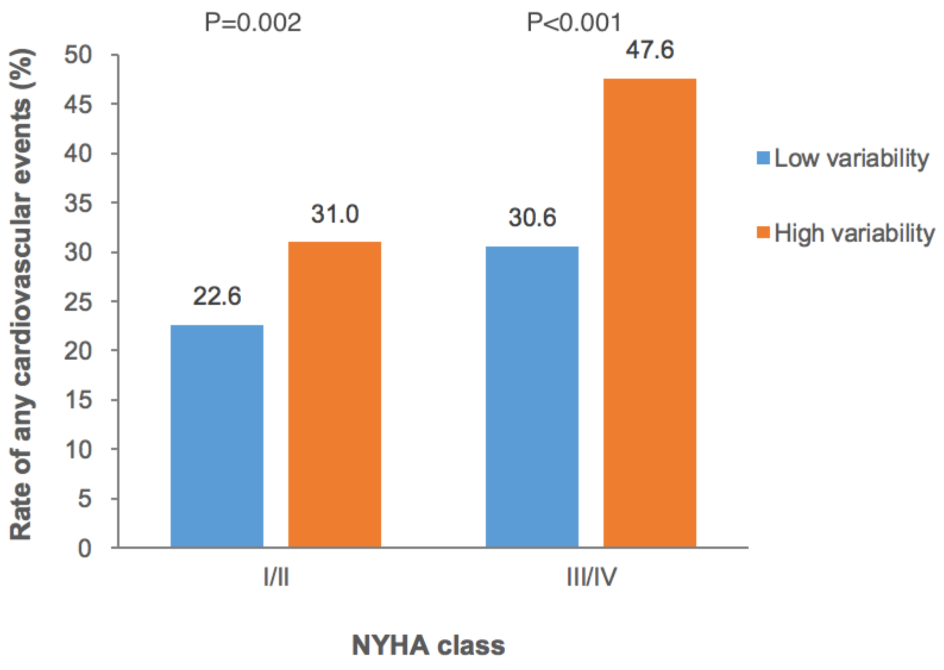

Figure S3c Body-weight variability and risk of any cardiovascular events by baseline BMI.

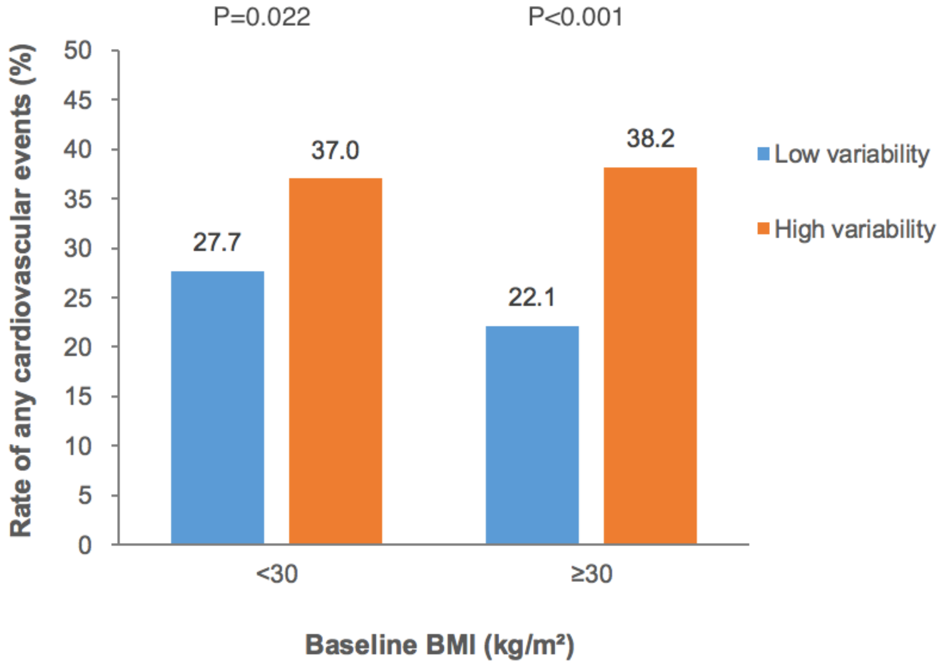

Figure S3d Body-weight variability and risk of any cardiovascular events by weight change direction.

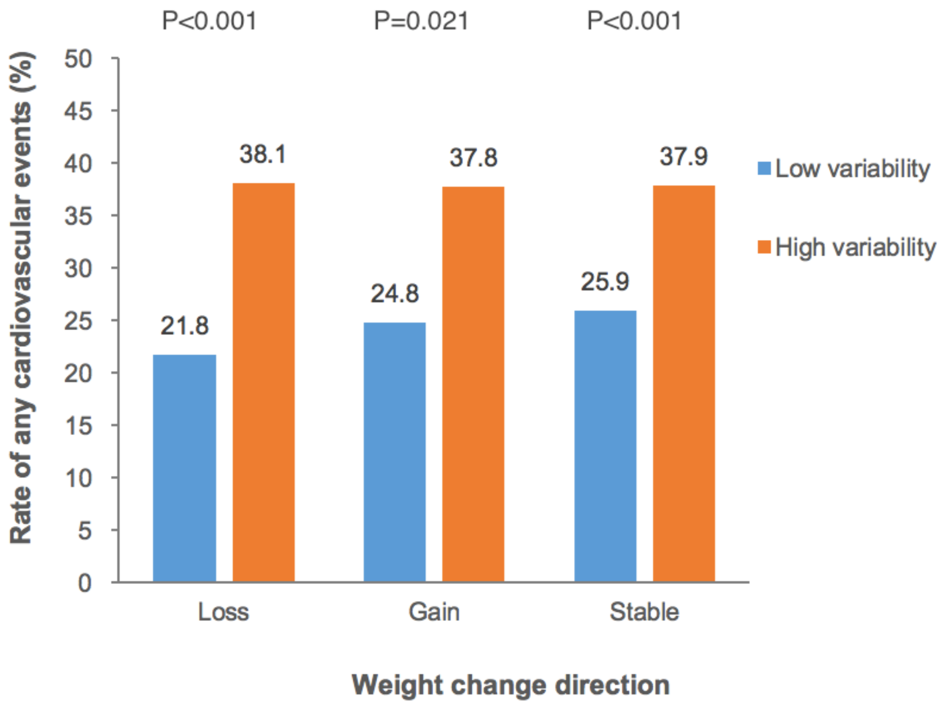

NYHA, New York Heart Association; BMI, body mass index;

Figure S3a-d indicates unadjusted models to compared rate of any cardiovascular events between patients of high versus low variability among subgroups.
